# Research on the influence of radiotherapy-related genes on immune infiltration, immunotherapy response and prognosis in melanoma based on multi-omics

**DOI:** 10.3389/fimmu.2024.1467098

**Published:** 2024-12-02

**Authors:** Yujing Shi, Wantong Zhao, Yuanjian Ding, Xiaolin Ge, Mengyang Ju

**Affiliations:** ^1^ Department of Oncology, Affiliated Jurong Hospital of Jiangsu University, Zhenjiang, ;China; ^2^ Department of Radiation Oncology, Affiliated Zhongshan Hospital of Dalian University, Dalian, ;China; ^3^ Department of Radiation Oncology, Osaka University Graduate School of Medicine, Osaka, ;Japan; ^4^ Department of Radiation Oncology, the First Affiliated Hospital of Nanjing Medical University, Nanjing, ;China

**Keywords:** skin cutaneous melanoma, radiotherapy, immunotherapy, tumor microenvironment, prognosis

## Abstract

**Background:**

Skin cutaneous melanoma (SKCM) is a significant oncological challenge due to its aggressive nature and poor treatment outcomes. This study explores the comprehensive effects of radiotherapy (RT) in SKCM, focusing on cell signaling pathways, immune infiltration, immune gene correlations, immunotherapy response, and prognosis.

**Methods:**

Using the Cancer Genome Atlas (TCGA) database, differentially expressed genes (DEGs) in SKCM patients undergoing RT were identified. A risk score model based on these DEGs was developed to assess the effects of RT-related genes on drug sensitivity, immune cell infiltration, immunotherapy response, and prognosis through multi-omics analysis. Human melanoma cells UACC62 and UACC257 were irradiated with 8 Gy gamma ray to establish an *in vitro* model, verifying the impact of radiotherapy on gene expression.

**Results:**

The risk score demonstrated significant prognostic value and emerged as an independent prognostic factor. miRNA-mRNA and transcription factor regulatory networks underscored its clinical significance. Four key genes were identified: DUSP1, CXCL13, SLAMF7, and EVI2B. Analysis of single-cell and immunotherapy datasets indicated that these genes enhance immune response and immunotherapy efficacy in melanoma patients. PCR results confirmed that gamma rays increased the expression of these genes in human melanoma cells UACC62 and UACC257.

**Conclusion:**

Using a multi-omics approach, we analyzed and validated the impact of RT on the immune landscape of melanoma patients. Our findings highlight the critical role of RT-related genes in predicting SKCM prognosis and guiding personalized therapy strategies, particularly in the context of immunotherapy. These contribute to understanding the role of radiotherapy combined with immunotherapy in melanoma.

## 1 Introduction

Skin cutaneous melanoma (SKCM), characterized by the malignant transformation of melanocytes, stands as a formidable challenge in oncology due to its aggressive nature and propensity for metastasis (1). Ethnic disparities are evident in the incidence of SKCM, generally are reported to be higher among Caucasians (2). However, recent studies have identified a troubling trend of rapidly increasing SKCM incidence rates of Asians, such as in China (3), emphasizing the imperative for heightened awareness and preventive measures. Despite advancements in early detection and treatment modalities, including surgery, chemotherapy, and immunotherapy, the management of advanced SKCM remains a significant clinical dilemma, as evidenced by poor treatment outcomes in clinical settings. Reports from multiple clinical trials reveal a five-year survival rate of less than 30% (4, 5). This underscores the urgent necessity for innovative treatment approaches and early detection strategies aimed at enhancing patient outcomes and survival rates.

Radiotherapy (RT) plays a critical role in controlling local disease, managing inoperable lesions, and improving overall survival rates, particularly in cases of unresectable or metastatic SKCM (6). The use of RT in SKCM management has evolved from a palliative approach to an integral component of multidisciplinary treatment strategies (7). With the development of immunotherapy, RT has been reported to enhance the effect of immune checkpoint inhibitors through abscopal effects (8), to achieve an additive anti-tumor effect in patients with metastatic melanoma while ensuring safety (9, 10).

Despite its therapeutic potential modulate tumor immunogenicity, challenges remain in optimizing the efficacy and safety of RT for SKCM. Additionally, the identification of predictive biomarkers to guide patient selection and treatment response represents a critical area of ongoing research.

In recent years, the field of bioinformatics has experienced significant advancements, particularly in the analysis of complex biological data. Bioinformatics has become an indispensable tool for interpreting high-throughput data, enabling researchers to identify key genetic components and elucidate their roles in various biological processes and diseases. In this study, we aimed to thoroughly investigate the role of RT in melanoma among the distribution of cell signaling pathways, immune infiltration, correlation of immune genes, sensitivity of chemotherapeutic drugs, and transcription factor regulation and develop a new prognostic model based on genes differentially associated with RT in melanoma by bioinformatical analyze and *in vitro* validation.

## 2 Materials and methods

### 2.1 Data downloads

We obtained the processed SKCM original expression data from TCGA database, which included a total of 285 melanoma patients: 247 patients in the “RT Not accepted” group and 38 patients in the “RT Accepted” group.

We downloaded the Series Matrix File data for GSE53118 (11) from the NCBI GEO public database, which is annotated under the platform GPL6884 and contains expression profile data for 69 patients. The MSKCC dataset (12) provides expression profile data for 140 patients.

Additionally, we downloaded the single-cell data file for GSE215120 (13) from the NCBI GEO public database, which includes single-cell expression profiles for four complete samples for single-cell analysis.

### 2.2 Differential expression analysis

The Limma package, an R software package for differential expression analysis (DEGs) of expression profiles, is used to identify significantly differentially expressed genes between groups (14). By using the “Limma” R package, we analyzed the molecular mechanisms underlying the data and identified differentially expressed genes between the two sample groups. The criteria for selecting differential genes were P Value < 0.05 and |logFC| > 0.585. Volcano plots and heatmaps of the differential genes were also generated.

### 2.3 Functional enrichment analysis

To understand the biological functions and signaling pathways involved in the differential genes, the “ClusterProfiler” R package was used for functional annotation, providing a comprehensive exploration of the functional relevance of these genes (15). Gene Ontology (GO) and Kyoto Encyclopedia of Genes and Genomes (KEGG) were used to evaluate related functional categories. GO and KEGG enrichment pathways with p-values and q-values less than 0.05 were considered significant categories.

### 2.4 Model construction and prognosis

Differentially related genes were selected, and a prognosis-related model was further constructed using lasso regression. A risk score formula was constructed for each patient by incorporating the expression values of each specific gene, weighted by the estimated regression coefficients from the lasso regression analysis. Based on the risk score formula, patients were divided into low-risk and high-risk groups using the median risk score as the cutoff. Survival differences between the two groups were assessed using Kaplan-Meier analysis, and comparisons were made using the log-rank test. Lasso regression analysis and stratified analysis were employed to evaluate the role of the risk score in predicting patient prognosis. The accuracy of the model predictions was studied using Receiver Operating Characteristic (ROC) curves.

### 2.5 WGCNA analysis

By constructing a weighted gene co-expression network, we identified co-expressed gene modules and explored the relationship between gene networks and risk scores, as well as the key genes within the network. Using the Weighted Correlation Network Analysis(WGCNA)-R package (16), we constructed a co-expression network for all genes in the SKCM dataset, screening the top 5000 genes by variance for further analysis, with a soft-thresholding power of 6. The weighted adjacency matrix was transformed into a topological overlap matrix (TOM) to estimate network connectivity, and hierarchical clustering was used to construct a clustering tree structure of the TOM matrix. Different branches of the clustering tree represented different gene modules, and different colors represented different modules. Genes were classified according to their expression patterns based on the weighted correlation coefficients, grouping genes with similar expression patterns into modules.

### 2.6 Immune cell infiltration analysis

The CIBERSORT method, widely used for evaluating immune cell types within the microenvironment, was applied (17). This method, based on the principle of support vector regression, performed deconvolution analysis of the expression matrix of immune cell subtypes. It includes 547 biomarkers, distinguishing 22 human immune cell phenotypes, including T cells, B cells, plasma cells, and myeloid subgroups. In this study, the CIBERSORT algorithm was used to analyze patient data to infer the relative proportions of 22 immune infiltrating cells and to analyze the correlation between gene expression levels and immune cell content.

### 2.7 Drug sensitivity analysis

Based on the largest pharmacogenomics database (GDSC, Genomics of Drug Sensitivity in Cancer, https://www.cancerrxgene.org/), we used the “oncoPredict” R package to predict the chemotherapy sensitivity of each tumor sample (18). The IC50 estimates for each specific chemotherapy drug treatment were obtained using regression methods, and 10-fold cross-validation was performed using the GDSC training set to test regression and prediction accuracy. All parameters were set to default values, including “combat” for batch effect removal and the average value for repeated gene expression.

### 2.8 GSVA analysis

Gene Set Variation Analysis (GSVA) is a non-parametric and unsupervised method for evaluating the enrichment of transcriptomic gene sets (19). GSVA scores gene sets of interest to translate gene-level changes into pathway-level changes, thereby determining the biological functions of the samples. In this study, gene sets were downloaded from the Molecular Signatures Database (version 7.0), and the GSVA algorithm was used to score each gene set comprehensively, assessing potential biological function changes in different samples.

### 2.9 GSEA analysis

Patients were divided into high-risk and low-risk groups based on the risk model, and GSEA was used to further analyze the differences in signaling pathways between the two groups. The background gene sets were downloaded from the MsigDB database and annotated as subtype pathways for differential expression analysis. Significantly enriched gene sets (adjusted p-value < 0.05) were ranked according to their enrichment scores. GSEA analysis is commonly used to explore the association between tumor subtypes and their biological significance.

### 2.10 Nomogram model construction

A nomogram is built on regression analysis, based on the expression levels of risk scores and clinical symptoms. It uses scaled line segments drawn on the same plane to express the relationships between variables in the predictive model. By constructing a multivariate regression model, scores were assigned to each level of influencing factors based on their contribution to the outcome variable (regression coefficient). The total score was then calculated by summing the individual scores, thereby estimating the predictive value.

### 2.11 miRNA network construction

miRNAs (MicroRNAs) are small non-coding RNAs that regulate gene expression by promoting mRNA degradation or inhibiting mRNA translation. We further analyzed whether certain miRNAs regulate the transcription or degradation of key genes. The miRNAs related to key genes were obtained from the miRcode database (20), and the miRNA network was visualized using Cytoscape software (21).

### 2.12 Transcriptional regulation analysis of key genes

This study used the “RcisTarget” R package to predict transcription factors. All computations by RcisTarget are based on motifs, and the normalized enrichment score (NES) of the motifs depends on the total number of motifs in the database. In addition to the motifs annotated by the source data, we inferred further annotations based on motif similarity and gene sequences. The first step in estimating the overexpression of each motif in the gene set is to calculate the area under the curve (AUC) for each motif-gene set pair, based on the recovery curve of motif ranking by the gene set. The NES of each motif was calculated from the AUC distribution of all motifs in the gene set.

### 2.13 Single-cell analysis

Initially, the expression profiles were imported using the Seurat package. Cells were filtered based on the total UMI count per cell, the number of genes expressed, and the percentage of mitochondrial and ribosomal reads in each cell. Outliers were defined as those deviating by three median absolute deviations (MAD) from the median, and cells with fewer than 200 detected genes were excluded. The filtering criteria were as follows: (nFeature_RNA > 200 & percent.mt <= 3MAD & nFeature_RNA <= 3MAD & nCount_RNA <= 3MAD & percent.ribo <= 3MAD), where nFeature_RNA represents the number of genes, nCount_RNA represents the total UMI count, percent.mt represents the percentage of mitochondrial reads, and percent.ribo represents the percentage of ribosomal reads. Subsequently, doublets were filtered using the DoubletFinder package, resulting in the retention of 28,371 cells.

The data were then normalized using the NormalizeData function. Cell cycle scores were computed using CellCycleScoring, and 2,000 highly variable genes were identified with the FindVariableFeatures function. The data were scaled with ScaleData to normalize them and to mitigate the effects of mitochondrial genes, ribosomal genes, and cell cycle on downstream analyses. Linear dimensionality reduction was performed using RunPCA. Batch effects were removed with Harmony, which iteratively clusters similar cells in PCA space across different batches while maintaining batch diversity within each cluster. Nonlinear dimensionality reduction was conducted using RunUMAP (Uniform Manifold Approximation and Projection), followed by identification of neighboring cells with FindNeighbors and clustering of cells into distinct cell clusters with FindClusters.

### 2.14 Immunotherapy response analysis

Tumor Immunotherapy Gene Expression Resource (TIGER) is a tumor immunotherapy gene expression resource, provides many useful modules for analyzing collected and user-provided data (22).

Key gene signatures were evaluated by the “gene set query” tab in “Immunotherapy Response” module (http://tiger.canceromics.org/#/immuneResponse).

### 2.15 Cell irradiation and quantitative real-time polymerase chain reaction

Human melanoma cell line UACC62 and UACC 257 were purchased from RIKEN (Saitama, Japan). Cells were cultured at 37°C in Roswell Park Memorial Institute (RPMI) 1640 medium and supplemented with 10% fetal bovine serum and a 1% penicillin-streptomycin solution purchased from Gibco (Albany, NY, USA) in a humidified incubator with 5% CO_2_.

To investigate the effects of radiation on the mRNA expression of key genes in human melanoma cells after RT, UACC62 and UACC257 cells were cultured in 6-cm dishes then irradiated with 8 Gy of Gamma ray radiotherapy (γ-RT).

γ-Ray radiation was conducted by a Γcell 40 Exactor (Best Theratronics Ltd., Ottawa, ON, Canada).

At 24 hours after RT, total RNA was isolated using a RNeasy Plus Mini kit (QIAGEN, Tokyo, Japan), and cDNA was synthesized using a qPCR RT Master Mix kit (TOYOBO, Osaka, Japan). PCR was performed using SYBR Green gene-expression assay with the Real-time PCR QuantStudio5 System (Thermo Fisher Scientific, London, UK). The gene-specific primer sequences used were as **Table 1**.

**Table 1:** Gene primer information.

Gene		5’->3’
DUSP1	Forward primer	GCCTTGCTTACCTTATGAGGAC
	Reverse primer	GGGAGAGATGATGCTTCGCC
SLAMF7	Forward primer	ACAACCCCTCTTGTCACCATA
	Reverse primer	CCCACATAGTAGATCCCTGAGTC
EVI2B	Forward primer	ACCAACACAATTCAGCGACAC
	Reverse primer	GTTGTAGGCAAGTGGTTGTCC

Glyceraldehyde 3-phosphate dehydrogenase (GAPDH) gene was used to normalize expression across assays and runs, and a cycle threshold (Ct) value for each sample was used to assess the expression level of genes.

### 2.16 Statistical analysis

Survival curves were generated using the Kaplan-Meier method and compared using the log-rank test. Multivariate analysis was performed using the Cox proportional hazards model. All statistical analyses were conducted using R (version 4.3.0), with p < 0.05 considered statistically significant.

## 3 Results

**Figure 1:**
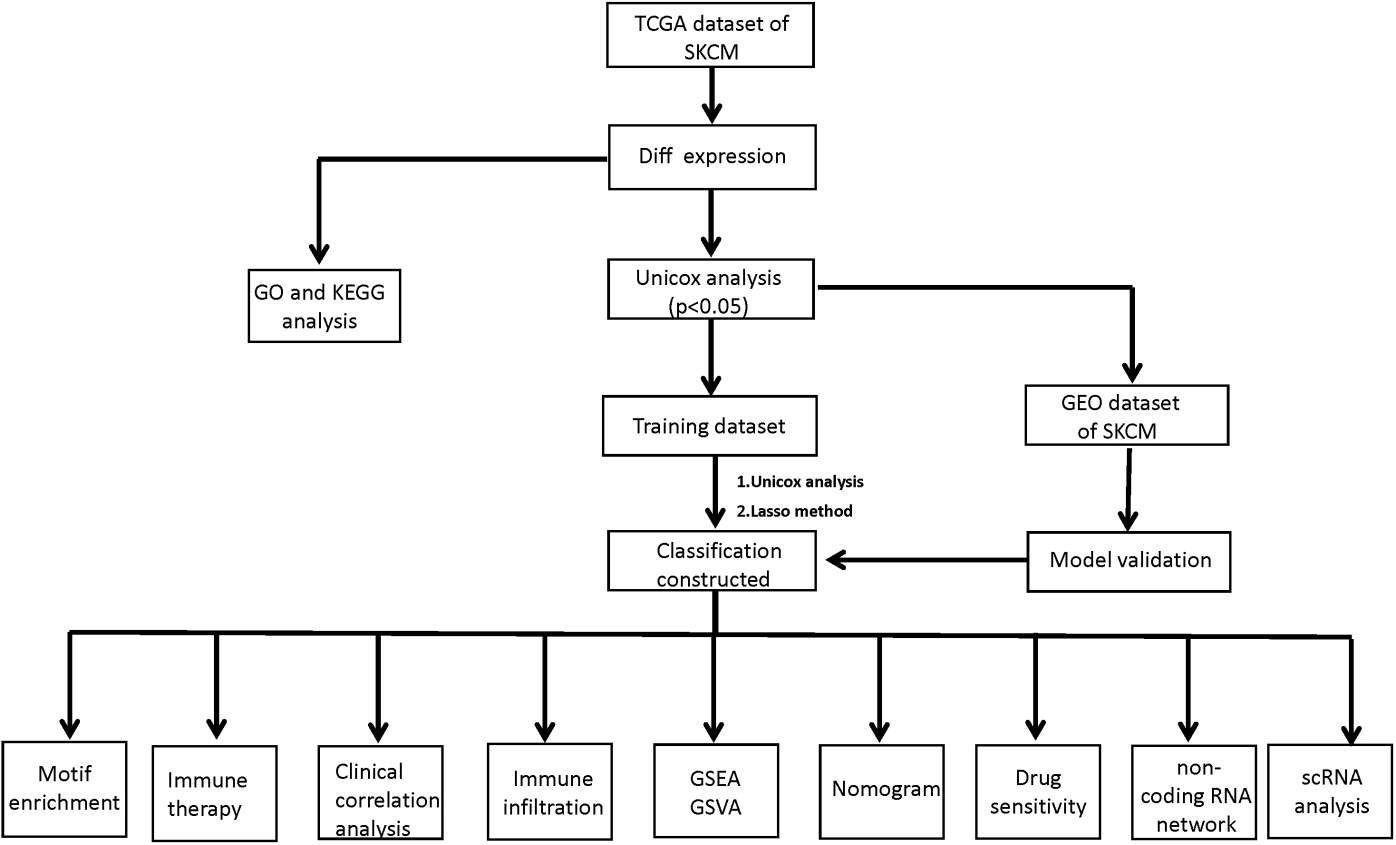
A workflow of the analysis protocol in the present study. TCGA, the Cancer Genome Atlas; SKCM, Skin Cutaneous Melanoma; GO, Gene Ontology; KEGG, Kyoto Encyclopedia of Genes and Genomes; GEO, Gene Expression Omnibus data base; Lasso, Least absolute shrinkage and selection operator; GSEA, Gene Set Enrichment Analysis; GSVA, Gene Set Variation Analysis.

**Figure 1** illustrates an analysis workflow of the present manuscript.

### 3.1 Identification of RT-related DEGs among SKCM patients

**Figure 2:**
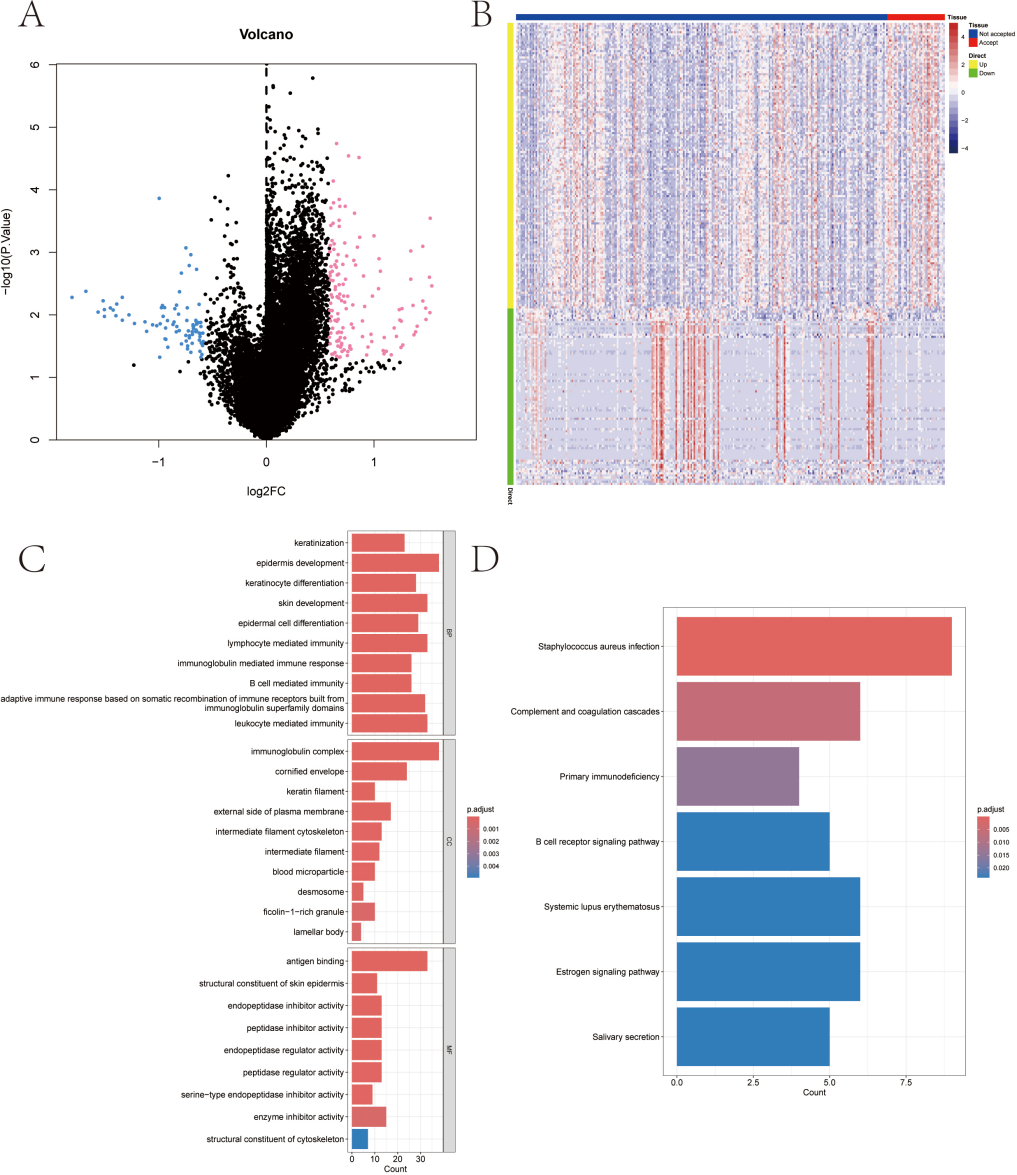
Radiotherapy-influenced genes among melanoma patients in TCGA-SKCM database and functional enrichment of differential genes. **(A, B)** Volcanic distribution plot and heat-map of differential genes. **(C)** Associated signaling pathways enriched in differential gene GO. **(D)** Associated signaling pathways enriched by differential gene KEGG.

We downloaded the SKCM dataset from the TCGA public database and extracted the data of 285 patients with melanoma, including 247 patients in the RT Not accepted group and 38 patients in the RT Accepted group. The limma package was used to screen the differentially expressed genes between the two groups. The screening conditions for differentially expressed genes were P Value < 0.05 and |logFC| >0.585. A total of 220 differentially expressed genes were screened out, including 136 up-regulated genes and 84 down-regulated genes (**Figures 2A, B**).

### 3.2 Results of functional enrichment of DEGs

In this study, the differentially expressed genes were further investigated for pathway analysis. GO results showed that the genes were mainly enriched in keratinocyte differentiation, epidermal cell differentiation, immunoglobulin complex and other pathways (**Figure 2C**). KEGG analysis showed that these differentially expressed genes were mainly enriched in Complement and coagulation cascades, B cell receptor signaling pathway and Estrogen signaling pathway and other pathways (**Figure 2D**).

### 3.3 Screening of prognostic genes and construction of RT-related risk score model in SKCM patients

**Figure 3:**
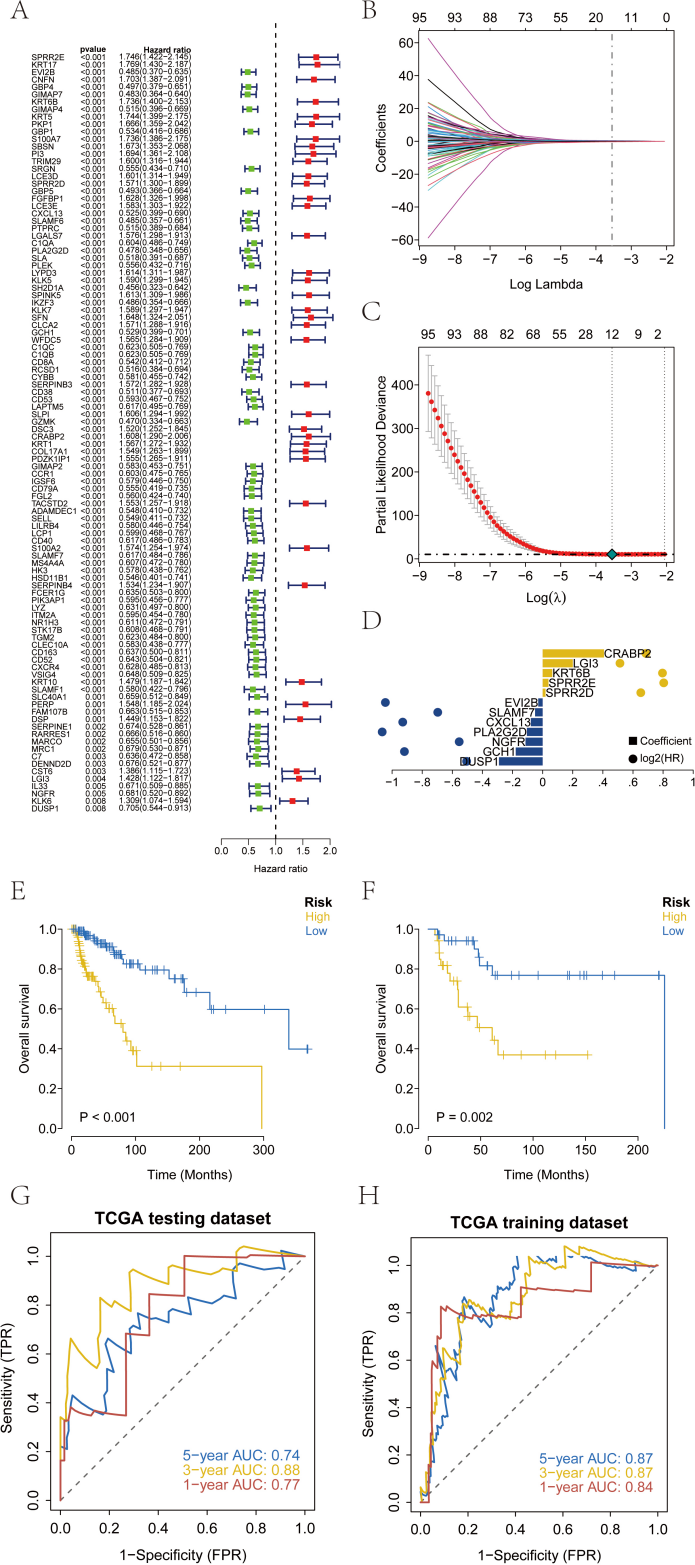
Construction and validation of risk scores based on patient prognosis information from TCGA-SKCM database. **(A)** A total of 102 genes related to prognosis were screened by Cox univariate regression. **(B-D)** The optimal riskscore value was obtained by lasso regression analysis. **(E)** Overall survival analysis of risk score in TCGA training database. **(F)** Overall survival analysis of risk score in TCGA testing database. **(G, H)** AUC of the risk score among training and testing database. AUC, areas under the curve.


The clinical information of SKCM patients was collected from TCGA database, and the prognostic genes in SKCM were screened by Cox univariate regression based on the differential genes. The results showed that a total of 102 prognostic related genes were screened by Cox univariate regression (p value< 0.01) (**Figure 3A**). According to the prognostic genes, the feature genes in SKCM were screened by lasso regression feature selection algorithm. The patients with survival data in the SKCM data set were randomly divided into training set and test set according to the ratio of 3:1. After lasso regression analysis, the best risk score value corresponding to each sample was obtained for subsequent analysis (**Figures 3B–D**). Therefore, we constructed the calculation formula and named it the RT-related risk score.

$$\begin{array}{l} RT-related\;risk\;score\;=DUSP1\times \left(-0.289869459740093\right)+GCH1\times \left(-0.178521004363843\right)+NGFR\times \left(-0.113723425288065\right)+PLA2G2D\times \left(-0.104760807889745\right)+CXCL13\times \\ \left(-0.0783756785513177\right)+SLAMF7\times \left(-0.0501776740893767\right)+EVI2B\times \left(-0.0289365802842518\right)+SPRR2D\times 0.0191166637996898+SPRR2\times 0.038490593757909+KRT6B\times \\ 0.0641695020289119+LGI3\times 0.200278253670939+CRABP2\times 0.407272355942318. \end{array}$$

The patients were divided into high-risk group and low-risk group according to the RT-related risk score, and Kaplan-Meier curve was used for analysis. The OS of the high-risk group was significantly lower than that of the low-risk group in both the training set and the test set (**Figures 3E, F**). In addition, the ROC curve results of the training set and the test set indicated that the model had good validation efficiency (**Figures 3G, H**).

**Figure 4:**
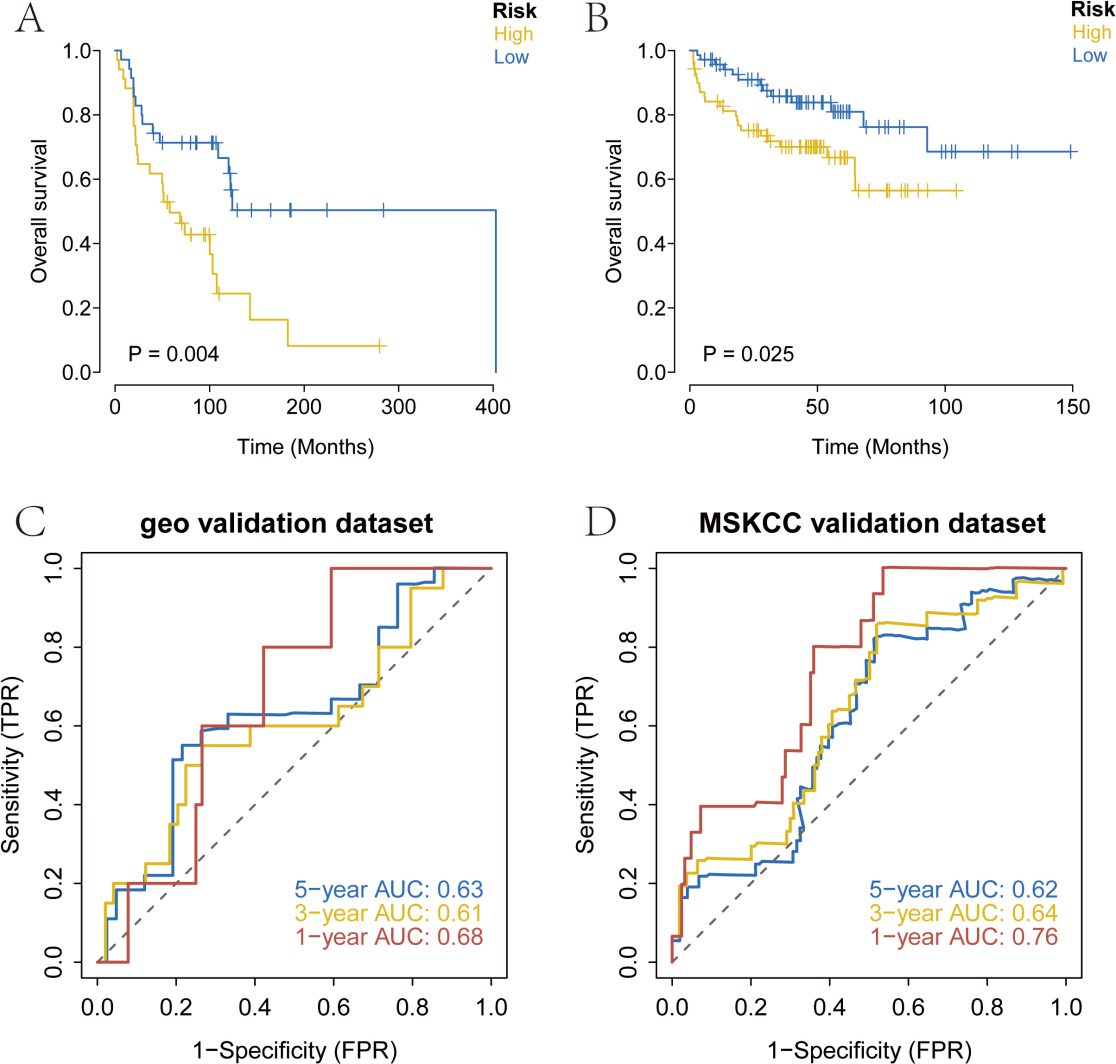
External validation of the risk score using prognosis data from melanoma patients in the GEO and MSKCC databases. **(A)** Overall survival analysis of risk score in GSE53118 database. **(B)** Overall survival analysis of risk score in MSKCC database. **(C, D)** AUC of the risk score among GEO and MSKCC database. MSKCC, Memorial Sloan-Kettering Cancer Center.

We downloaded the survival data of SKCM patients from the GEO database (GSE53118) and the processed survival data from the MSKCC database. Using our model, we analyzed the clinical classification of patients and evaluated the survival differences between the two groups using Kaplan-Meier analysis to assess the stability of the prediction model. The results indicated that the overall survival (OS) of the high-risk group was significantly lower than that of the low-risk group in both the GEO and MSKCC external validation sets (**Figures 4A, B**). To verify the accuracy of the model, we conducted ROC curve analysis using external datasets. The results demonstrated that the model had strong predictive efficiency for patient prognosis (**Figures 4C, D**).

### 3.4 WGCNA analysis revealed the RT-related risk score related regulatory network and screening of key genes in SKCM

**Figure 5:**
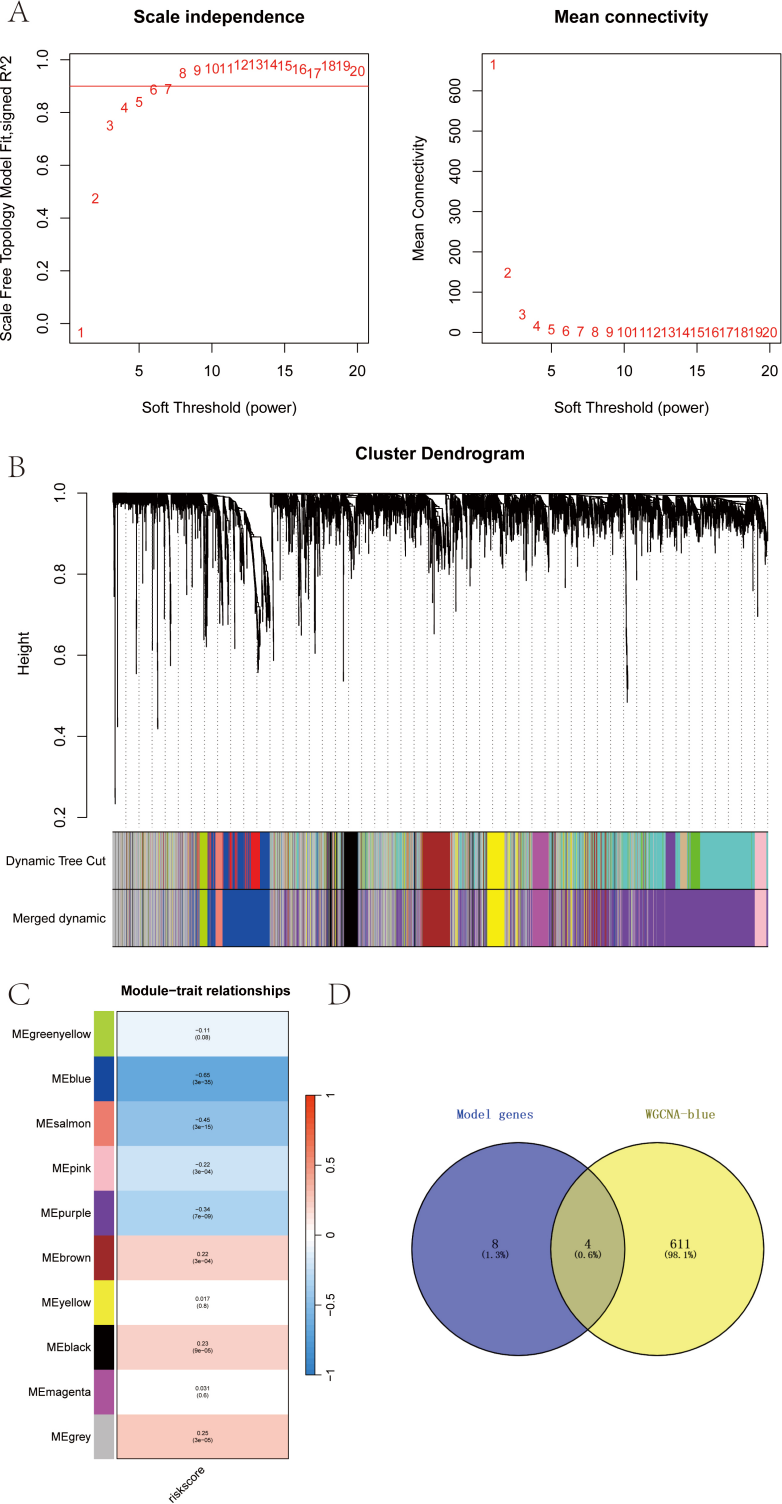
**(A-D)** WGCNA analysis revealed the RT-related risk score related regulatory network and screening of key genes in SKCM.

Based on the SKCM data, we further constructed a WGCNA network to explore the regulatory network associated with the RT-related risk score in SKCM. Here, the modeling genes with higher correlation with the risk score of the model were selected as the key genes by WGCNA analysis. The soft threshold β was set to 6 (**Figure 5A**), and gene modules were detected based on the TOM matrix. Ten gene modules were identified in SKCM (**Figure 5B**), namely black (223 genes), blue (615 genes), brown (361 genes), greenyellow (112 genes), grey (1253 genes), magenta (135 genes), pink (163 genes), purple (1801 genes), salmon (56 genes), and yellow (281 genes). Further analysis of the correlation between modules and traits revealed that the blue module had the highest correlation with the RT-related risk score (cor = -0.65, p = 3e−35) (**Figure 5C**). Subsequently, we intersected the genes in the blue module with the model genes, resulting in four intersecting genes (**Figure 5D**). These four genes, which will be the focus of our subsequent research, are DUSP1, CXCL13, SLAMF7, and EVI2B. Using the ROC curve, with whether the patient accepted radiotherapy as the analysis condition, the identification value of four key genes for radiotherapy in melanoma patients was analyzed (**Supplementary 1**).

### 3.5 Verification of the applicability and significance analysis of the risk score in clinical typing of SKCM samples

**Figure 6:**
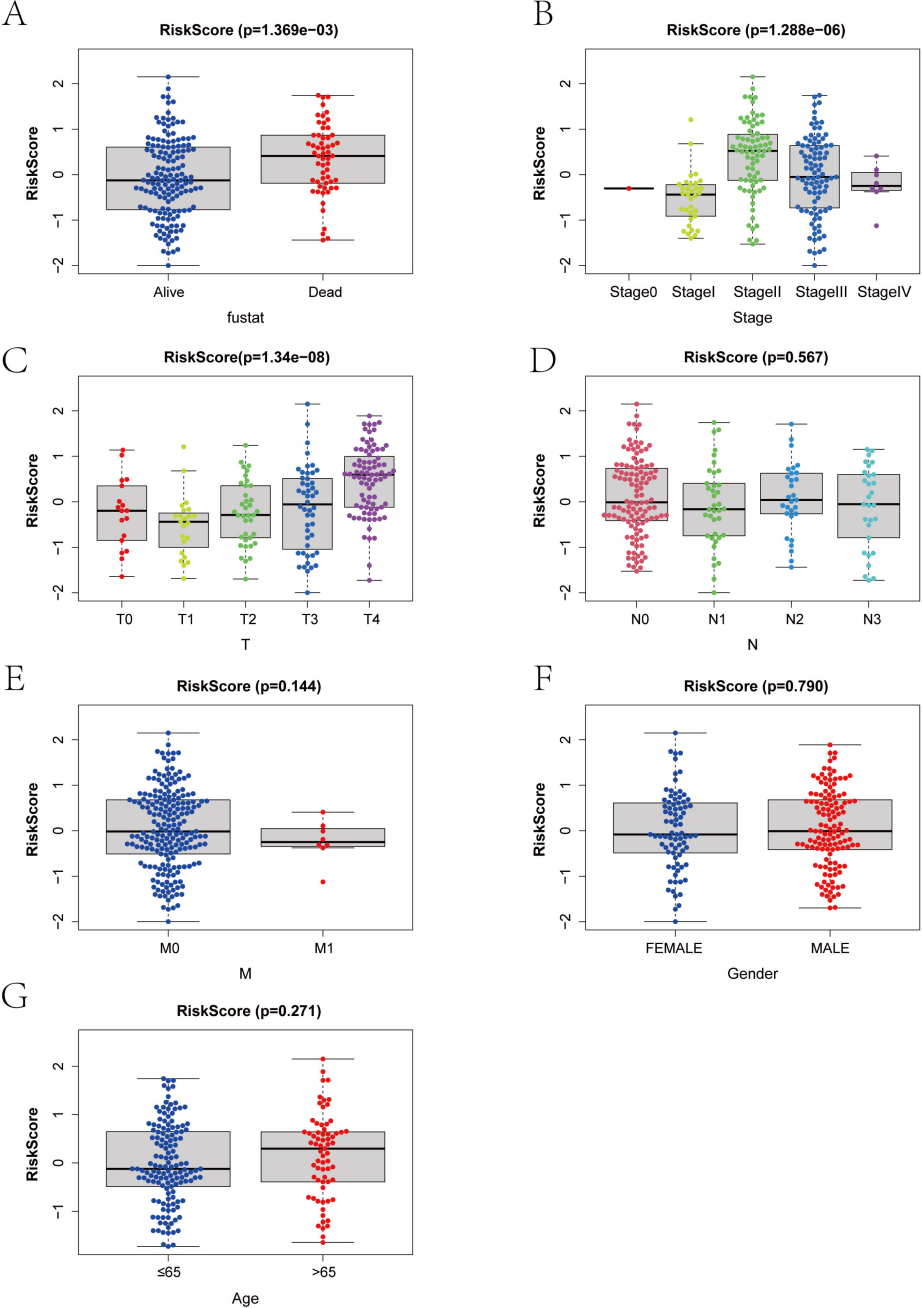
**(A–G)** Correlation between risk score and clinical index in melanoma patients. T, Tumor; N, Node; M, Metastasis.

We categorized the RT-related risk score values of the samples based on clinical indicator values and presented the results of each clinical indicator grouping using box plots (**Figure 6**). A rank sum test was performed, revealing that the distribution of RT-related risk score values differed significantly between groups for the clinical indicators of Stage, T, and Fustat (p < 0.05). This finding demonstrates that the RT-related risk score obtained from our modeling analysis has good applicability for classifying SKCM samples.

### 3.6 Analysis of the impact of risk score on tumor immune infiltration and regulatory factors

By analyzing the relationship between RT-related risk score and tumor immune infiltration, we aimed to further explore the potential molecular mechanisms by which RT-related risk score affects the progression of SKCM.

**Figure 7:**
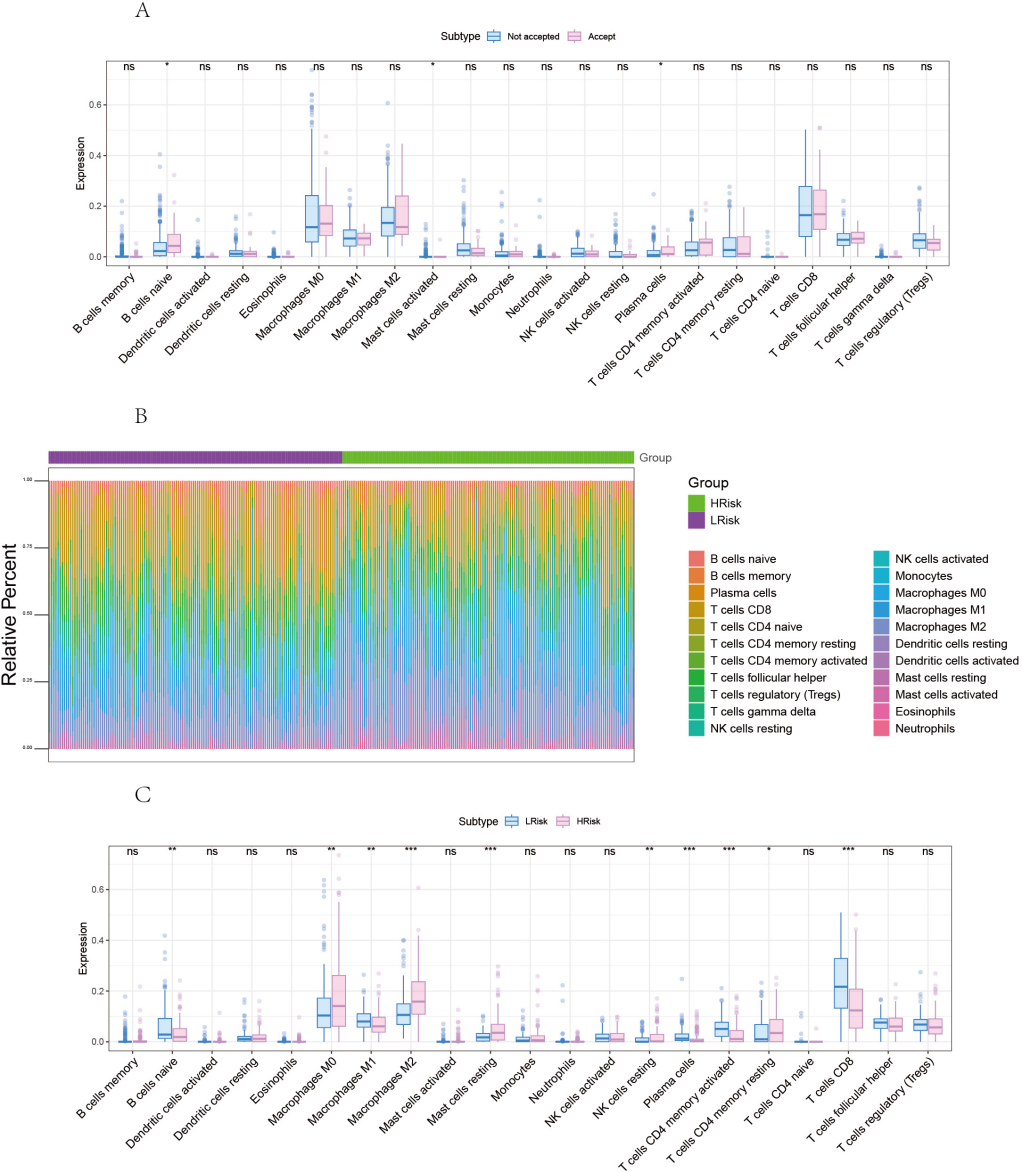
Correlation between risk score and tumor microenvironment in melanoma. **(A)** Comparison of differences in immune cells between radiotherapy *Accepted* and *Not Accepted* groups. **(B)** Overview of the differences in immune cell content between high and low-risk groups. **(C)** Comparison of differences in immune cells between high and low-risk groups. *p<0.05, **p<0.01, ***p<0.001 ns, No Significant.

First, we analyzed the effect of radiotherapy on immune cell infiltration. We then compared the differences in immune cell infiltration between high-risk and low-risk groups (**Figure 7**).

**Figure 8:**
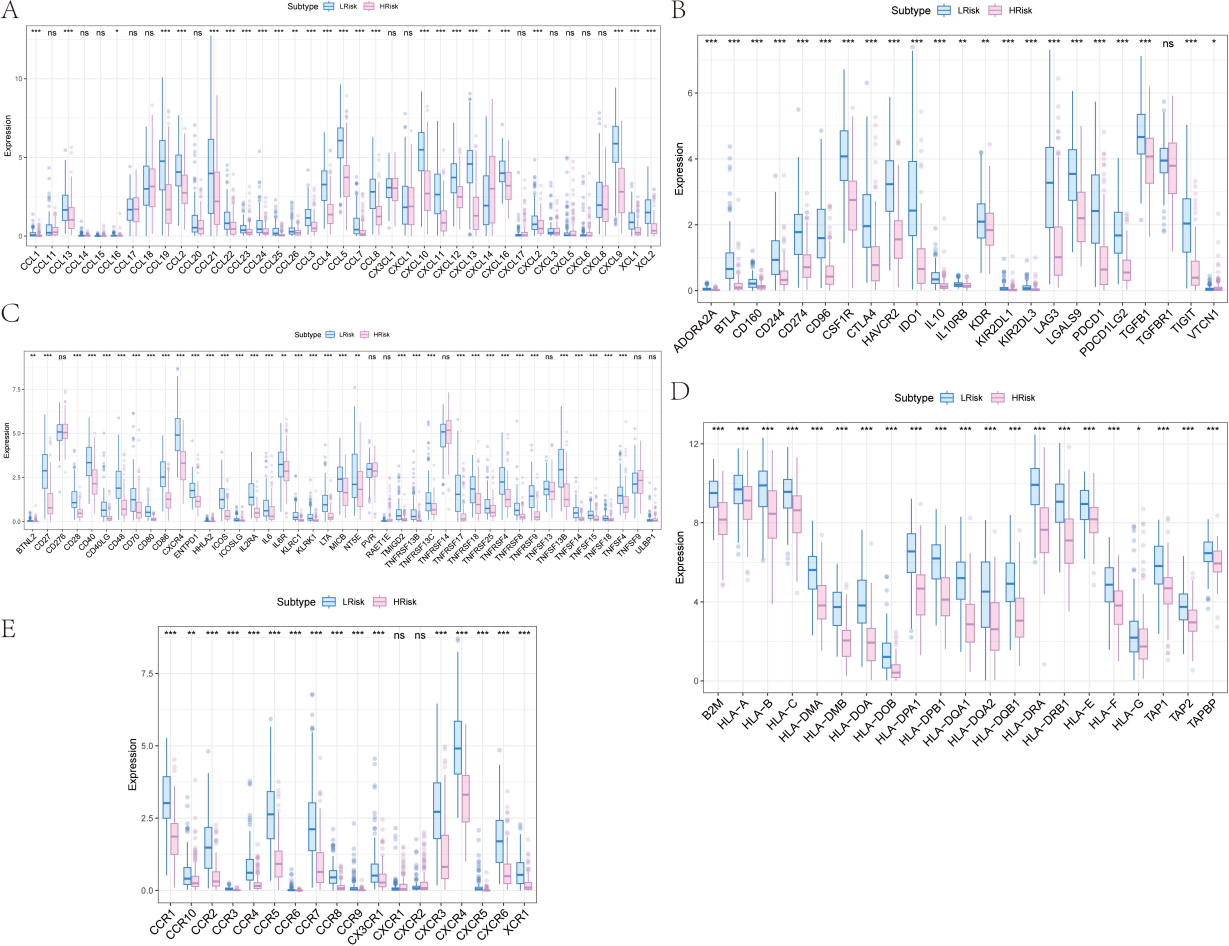
Differences of immune regulatory factors between the high and low risk groups. **(A)** Association of risk score with chemokines. **(B)** Correlation of risk score with immunosuppressive agents. **(C)** Association of risk score with immune agonists. **(D)** Association of risk score with MHC. **(E)** Association of risk score with MHC receptors. MHC, major histocompatibility complex. *p<0.05, **p<0.01, ***p<0.001 ns, No Significant.

The proportion of immune cell content between high and low-risk groups is illustrated. Our study further compared the immune cell content differences between low-risk and high-risk groups, revealing that in the high-risk group, B cells naive, Macrophages M1, Plasma cells, T cells CD4 memory activated, and T cells CD8 were significantly reduced, whereas Macrophages M0, Macrophages M2, Mast cells resting, and T cells CD4 memory resting were significantly increased. We further obtained various categories of immune regulatory genes from the TISIDB database (http://cis.hku.hk/TISIDB/), including immunosuppressive factors, immune-stimulating factors, chemokines, major histocompatibility complex, and chemokine receptors (**Figure 8**).

### 3.7 Analysis of the impact of risk score on chemotherapy drug sensitivity in skcm patients

**Figure 9:**
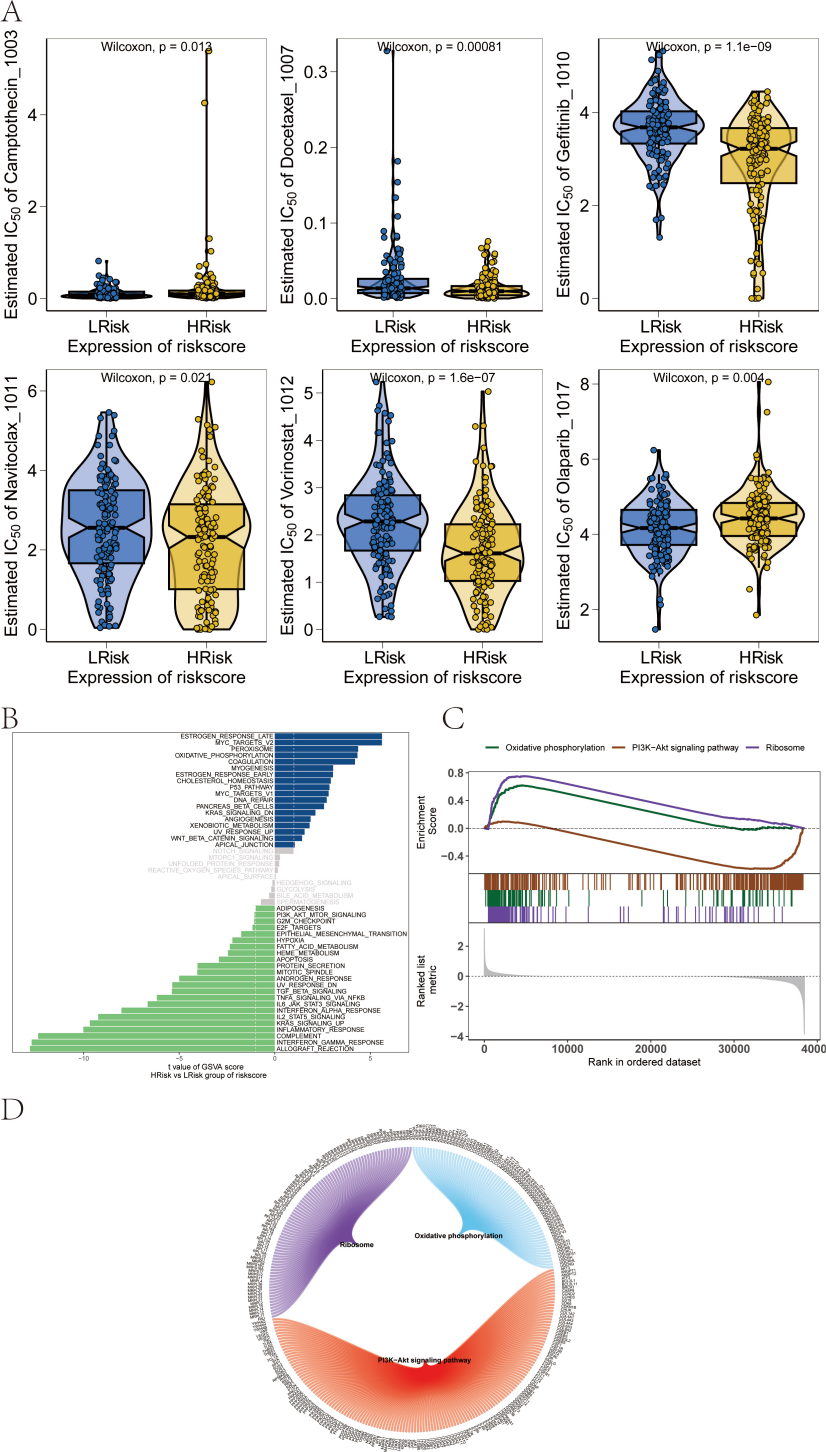
Influence of drug sensitivity and discussion on specific signaling mechanisms related to risk score. **(A)** Relationship between risk score and drug sensitivity in melanoma. **(B)** Results from GSVA. **(C)** Results from GSEA. **(D)** Network of molecular interactions between pathways.

Our study is based on the drug sensitivity data from the GDSC database, using the R package “oncoPredict” to predict the chemotherapy sensitivity of each tumor sample, and further explore the relationship between RT-related risk score and the sensitivity to common chemotherapy drugs. Research results show that the level of RT-related risk score is significantly related to the patient’s sensitivity to Camptothecin_1003, Docetaxel_1007, Gefitinib_1010, Navitoclax_1011, Vorinostat_1012, and Olaparib_1017 (**Figure 9A**).

### 3.8 Potential molecular mechanisms of risk score on tumor progression in skcm patients

We next studied the specific signaling pathways involved in high and low-risk related models and explored the potential molecular mechanisms by which RT-related risk scores affect tumor progression. The GSVA results showed that the differential pathways between the two groups of patients were mainly enriched in signaling pathways such as MYOGENESIS, P53_PATHWAY, and DNA_REPAIR (**Figure 9B**). GSEA results showed that the pathways involved were Ribosome, Oxidative phosphorylation, and PI3K−Akt signaling pathway (**Figure 9C**). The molecular interaction network between each pathway is shown in the figure (**Figure 9D**).

### 3.9 Analysis of risk score as an independent prognostic factor and validation of the predictive model for SKCM patients

**Figure 10:**
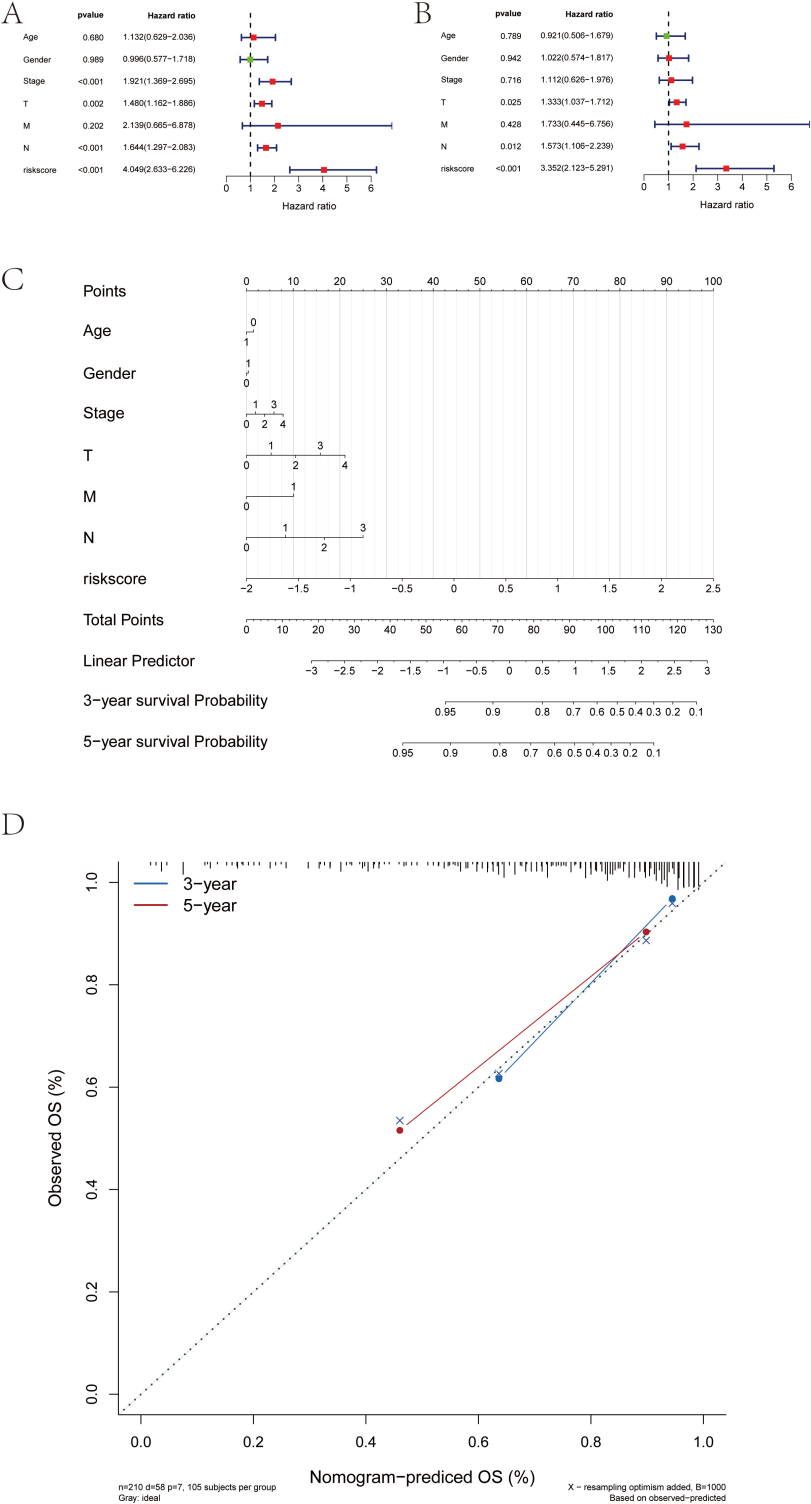
Construction of the risk score-associated nomogram prediction model and clinical relevance analysis. **(A, B)** Risk score is an independent prognostic factor for SKCM patients. **(C)** Risk score grade value of nomogram prediction model has significant contribution in the process. **(D)** Calibration curves of nomogram for predicting OS at 3-year and 5-year.

We found that RT-related risk score is an independent prognostic factor in patients with SKCM through univariate and multivariate analyses (**Figures 10A, B**). Samples were then divided into high-risk and low-risk groups according to the median RT-related risk score value, and the results of their regression analysis were displayed in the form of a nomogram. The results of the logistic regression analysis showed that in all our samples, the RT-related risk score value significantly contributed to the scoring process of the nomogram prediction model (**Figure 10C**). Additionally, we conducted prediction analyses for the three-year and five-year periods of melanoma (**Figure 10D**).

### 3.10 miRNA-mRNA network and transcription factor regulatory mechanism analysis

**Figure 11:**
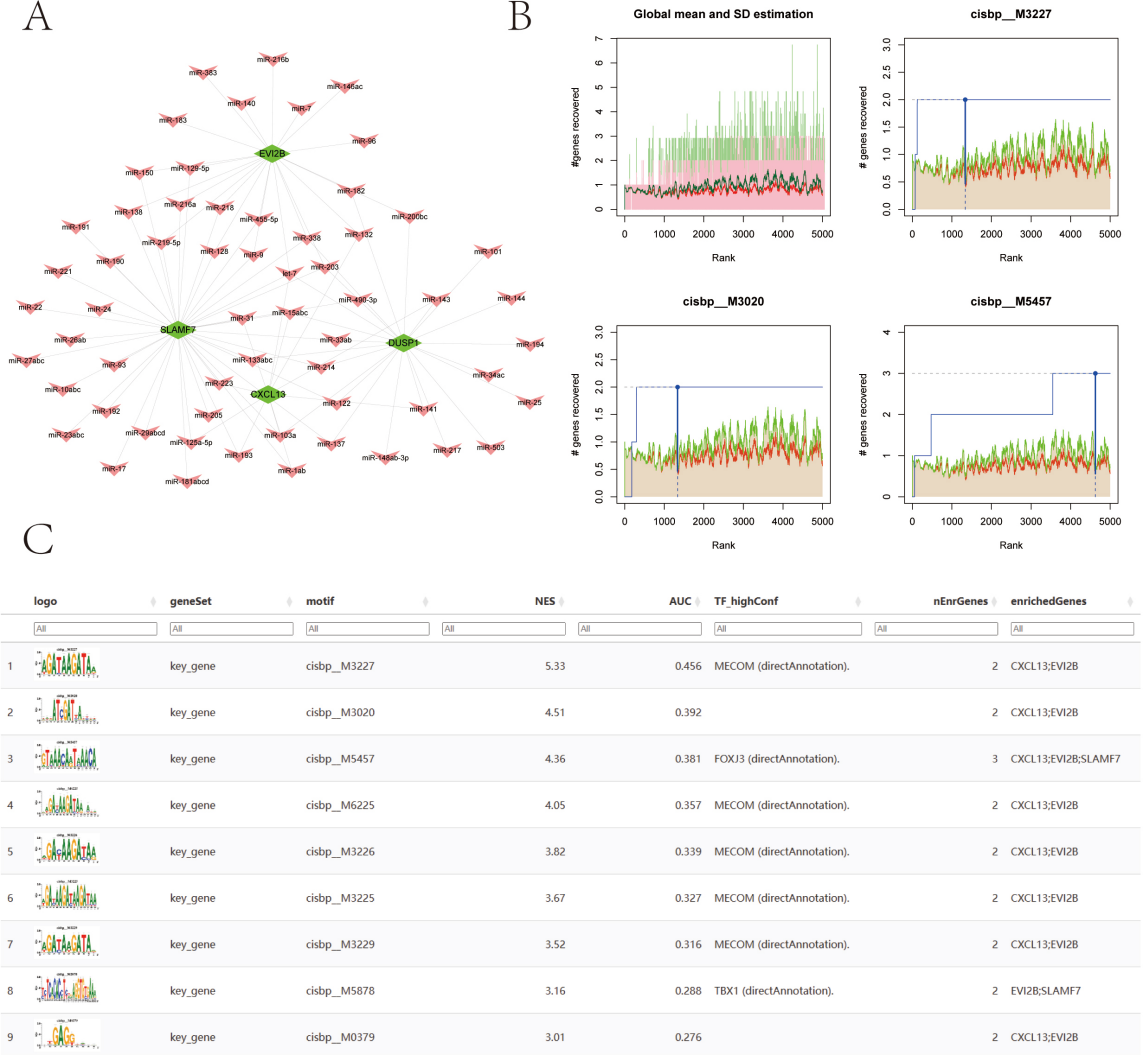
Transcriptional regulatory network of 4 key genes.

We used the miRcode database to perform reverse prediction on 4 key genes, resulting in 60 miRNAs and a total of 95 mRNA-miRNA pairs, which were visualized using Cytoscape (**Figure 11A**). We then used these four key genes as the gene set for this analysis and found that they are regulated by common mechanisms involving multiple transcription factors. Consequently, enrichment analysis of these transcription factors was performed using cumulative recovery curves. Motif-TF annotation and selection analysis of important genes showed that the Motif with the highest normalized enrichment score (NES: 5.33) is cisbp:M3227. All enriched motifs and corresponding transcription factors of model genes are displayed (**Figures 11B, C**).

### 3.11 Cell subtype clustering, cell type annotation, and key gene expression analysis from single-cell data

**Figure 12:**
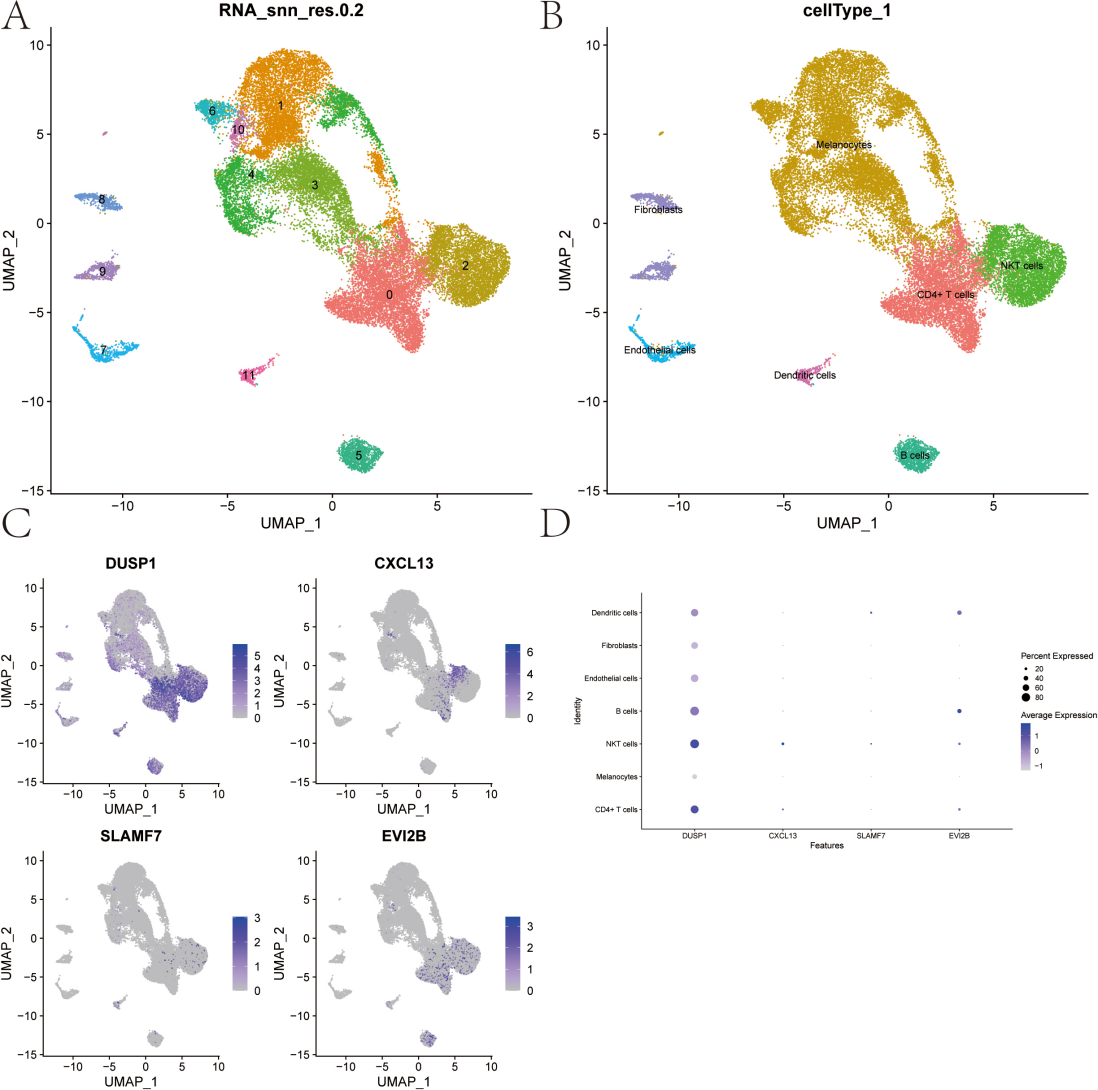
Cell Subtype Clustering, Cell Type Annotation, and Key Gene Expression Analysis from Single-cell data. **(A)** UMAP clustering of single-cell RNA-seq data from 4 samples (GSE215120) downloaded from the NCBI GEO public database, resulting in 12 distinct cell subtypes. **(B)** Annotation of the 12 clusters using the SingleR R package, categorizing them into 7 cell types. **(C, D)** Expression levels of 4 key genes across the 7 identified cell types.

The single-cell data file GSE215120 was downloaded from the NCBI GEO public database, encompassing four single-cell samples, and all of them were marked as cutaneous melanoma: GSM6622299, GSM6622300, GSM6622301 and GSM6622302. Cell clustering was performed using the UMAP algorithm, resulting in 12 subtypes (**Figure 12A**). Annotation of each subtype was conducted with the R package SingleR, and the 12 clusters were categorized into seven cell types: CD4+ T cells, Melanocytes, NKT cells, B cells, Endothelial cells, Fibroblasts, and Dendritic cells (**Figure 12B**). The expression of four key genes was analyzed across these seven cell types. The results revealed that the DUSP1 gene was significantly expressed in CD4+ T cells and NKT cells; the CXCL13 gene was significantly expressed in CD4+ T cells and NKT cells; the SLAMF7 gene was significantly expressed in NKT cells and Dendritic cells; and the EVI2B gene was significantly expressed in B cells and Dendritic cells. These findings explore their potential impacts on immunotherapy and cell function (**Figures 12C, D**). Dot plot displaying marker genes of each cell type was showed in **Supplementary 2**.

### 3.12 Immunotherapy response analysis of key genes signature in melanoma patients.

The melanoma immunotherapy dataset PRJEB23709(n=91), GSE100797(n=25), GSE78220(n=28), GSE91061(n=109), Nathanson_2017(n=24), phs000452(n=153) were included to analyze the influence of DUSP1, CXCL13, SLAMF7 and EVI2B in immunotherapy response. Patients with partial response (PR) and complete response (CR) were included in the Responder Group (R), the patients with stable disease (SD) and progressive disease (PD) were included in the Non-Responder (NR).

**Figure 13:**
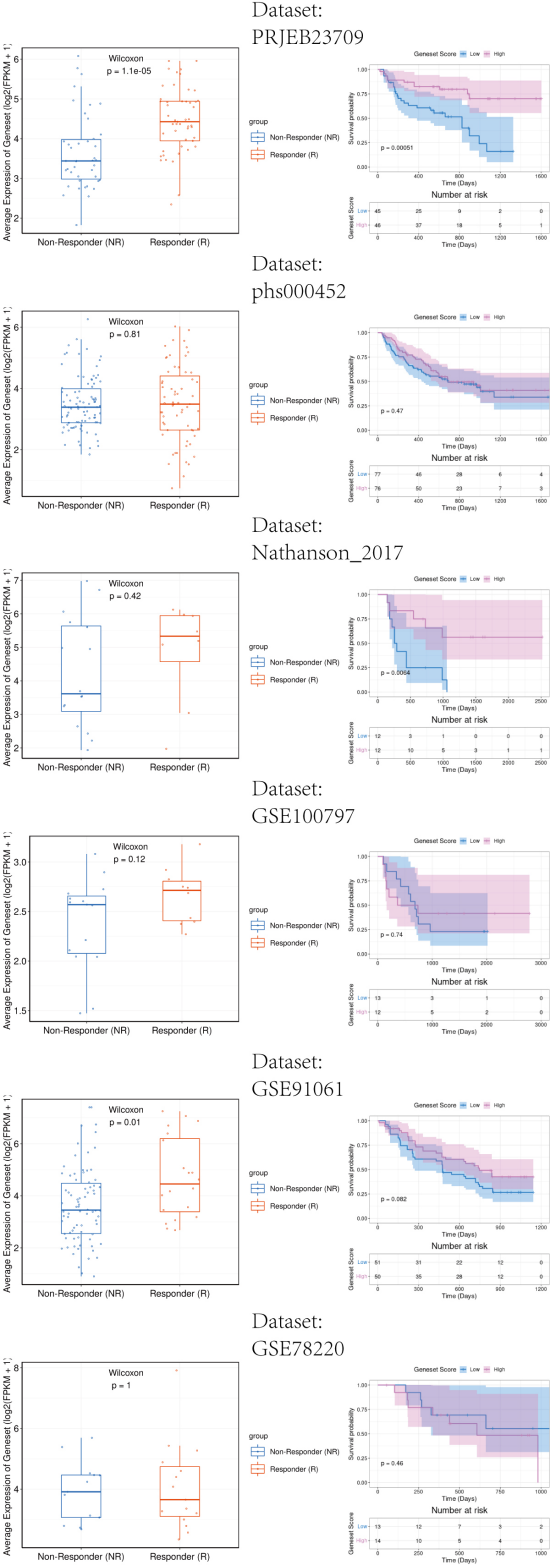
Immunotherapy response analysis of key genes signature in melanoma patients. Left side of the image: The relationship between gene expression and response to immunotherapy. The right side of the image: The relationship between gene expression and patient survival rates.

**Figure 13** showed that patients with high expression of the key genes signature exhibit a significantly better response to immunotherapy among GSE91061 and PRJEB23709. Survival analysis results indicate that high expression of the key genes is significantly associated with longer survival among Nathanson_2017 and PRJEB23709. These findings demonstrate that the key genes we have identified potentially enhance the response of melanoma patients to immunotherapy. This contributes to a better understanding of the positive role of radiotherapy in the immunotherapy of melanoma patients.

### 3.13 Validation of key genes expression by qPCR

**Figure 14:**
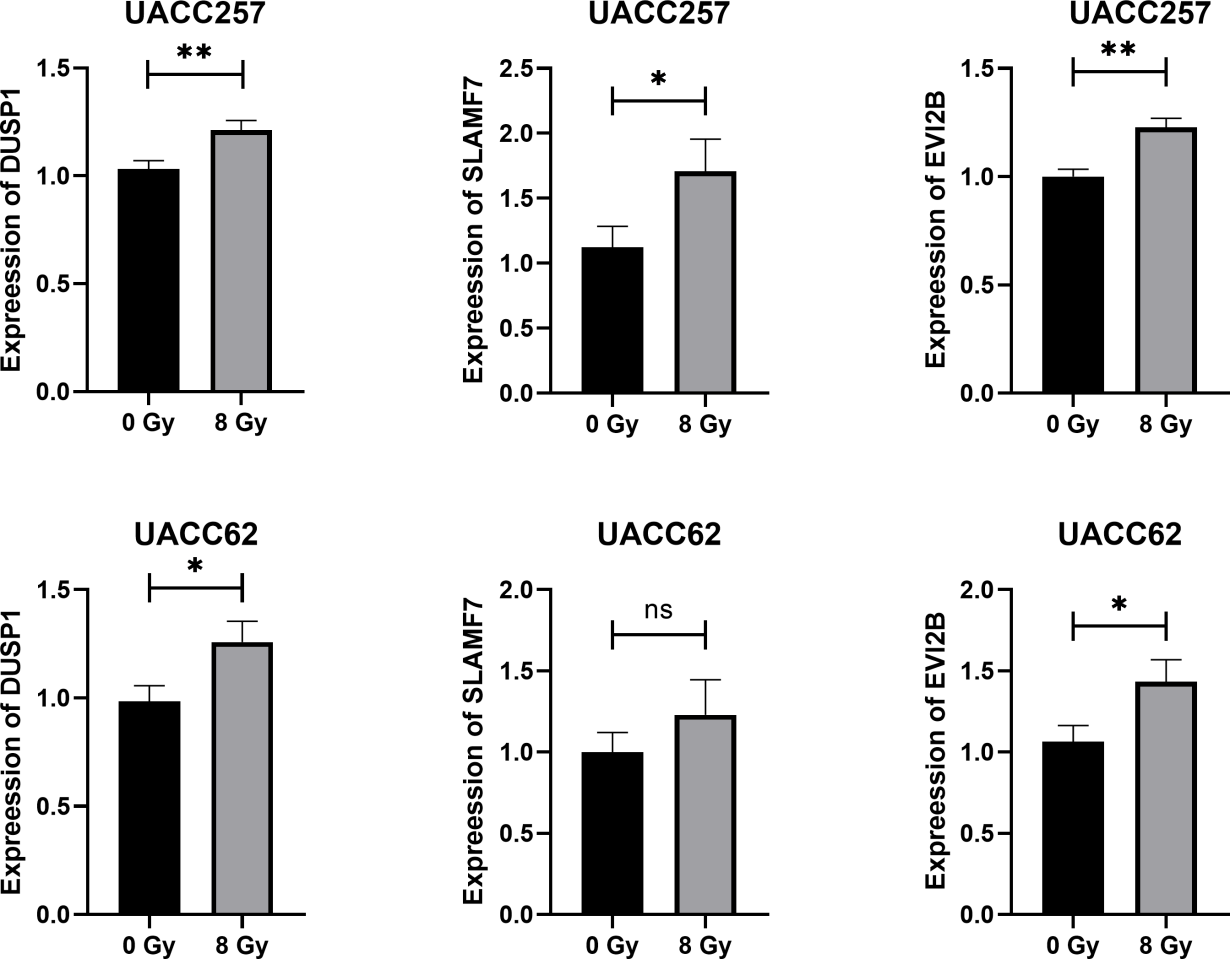
Results of qRT-PCR detection of key genes in human melanoma cell UACC62 and UACC257 after 8 Gy Gamma-ray radiation. *p<0.05, **p<0.01.

To further verify the regulation of key genes in melanoma cells by RT, human melanoma cells UACC62 and UACC257 were irradiated with 8 Gy gamma ray. At 24 h after irradiation, cellular RNA was extracted and analyzed by qPCR. The results in **Figure 14** showed that, except for SLAMF7 gene in UACC62 cells, the expression of other key genes in both cell lines was effectively increased by radiation. This further validates the regulation of key genes in melanoma by radiotherapy.

## 4 Discussion

Melanoma is an immunogenic tumor with multiple types of tumor-infiltrating lymphocytes (TILs) in the TME (23). Previous studies have confirmed that RT is closely related to influence tumor immunotherapy and modulate TME status (24, 25). However, the underlying mechanism seems unclear. Although the combination of radiotherapy and immunotherapy provides an opportunity to enhance the immunostimulatory effects of radiotherapy, the factors that affect the overall balance of immunomodulation are largely unknown (26). At the same time, few studies have focused on the comprehensive analysis of radiotherapy-related genes on immunotherapy response, immune cell infiltration and prognosis of melanoma patients.

In this study, we constructed a risk score model based on RT-related genes involved in melanoma patients using data from the TCGA-SKCM database. Validation results from different databases showed that the risk score model could effectively and reliably predict the prognosis of melanoma patients.

We investigated this RT-related risk score model in relation to clinical parameters and found that it was significantly associated with Stage, T, and Fustat. The results show a significantly elevated risk score in stage II melanoma patients. The American Joint Committee on Cancer (AJCC) introduced the 8th edition of the melanoma staging system, which is currently widely used worldwide (27). However, we have observed that in recent years, this staging system has been increasingly challenged, particularly in stage II subtypes with high-risk factors, such as stages IIB and IIC. Real-world clinical data indicate that the survival rates of IIC melanoma patients are lower than stage IIIA and IIIB patients (28, 29). Possible reasons include the higher risk of metastasis and recurrence in stage IIB and IIC patients (30, 31), as well as differences in immune cell infiltration (32). It aligns with our finding of the predictive effect of risk score and suggesting that the current AJCC8 staging system limits in accurately reflecting an increasing risk of mortality in melanoma patients.

Furthermore, we constructed a nomogram model to verify that the RT-related risk score is an independent prognostic factor among melanoma patients. This model allowed us to predict 3- and 5-year survival rates, indicating that the nomogram has good predictive efficacy and can guide clinical practice. We also analyzed the risk score model for chemotherapy drug sensitivity to further clarify its value in the comprehensive treatment of melanoma patients. Additionally, we used Cytoscape to construct a comprehensive transcriptional regulatory network of RT-related genes involved in radiotherapy in melanoma.

This RT-related risk score model, identified through comprehensive bioinformatics analyses, implicates various aspects of melanoma biology, including cell proliferation, apoptosis, and metastasis. The RT-related risk score derived from our model correlates with specific signaling pathways known to influence melanoma progression. For instance, pathways such as MYOGENESIS, P53, and DNA_REPAIR were differentially enriched in high-risk and low-risk groups, suggesting their potential role in modulating tumor behavior. Further exploration of these pathways could uncover novel therapeutic targets and improve our understanding of melanoma pathogenesis.

Immunotherapy is a critical treatment for patients with advanced SKCM (33, 34). Understanding the interaction between immune cells and melanoma can provide insights into the mechanisms driving tumor immune evasion and resistance to immunotherapy. The immune activity and tumor microenvironment (TME) status play a decisive role in the effectiveness of immunotherapy (35). Recent advances in tumor immunology have underscored the importance of immune infiltration in melanoma progression and treatment response (36). Immune cells within the TME, including T cells, macrophages, and dendritic cells, significantly influence tumor growth, either promoting or inhibiting it. The degree and type of immune infiltration are associated with clinical outcomes, with a high presence of cytotoxic T cells generally correlating with a better prognosis (37).

In clinical practice, various modalities of radiotherapy, such as the type of radiation, dose, and irradiation site, exert distinct influences on the efficacy of combined immunotherapy (38). Our previous *in vitro* experimental results indicated that B16F10 cells pretreated with melatonin exhibited significant differences in immune-related pathways after irradiation with varying doses of carbon ions compared to control cells, suggesting a potential to enhance the response to immunotherapy (39). Previous studies have demonstrated that the application of low-dose radiotherapy can effectively counteract the immunosuppressive effects within the tumor microenvironment, thereby promoting the infiltration of CD8+T cells (40). Additionally, it has been suggested that administering radiation to multiple sites within a tumor may activate a greater number of shared tumor-associated antigens, particularly in light of the heterogeneity observed across various tumor lesions (41). In this study, we have compiled information regarding the irradiation sites and patterns, as well as corresponding risk stratification, from the TCGA data collected, which is presented as **Supplementary 3**.

Our research focuses on characterizing the immune landscape of melanoma based on RT-related genes to develop predictive biomarkers and novel immunotherapeutic strategies. Our results found that the risk score had a significant effect on immune infiltration in melanoma patients. Furthermore, four key genes were identified: DUSP1, CXCL13, SLAMF7 and EVI2B. Findings based on single-cell data and melanoma immunotherapy datasets highlight the potential of these key genes in modulating immunogenicity and response to immunotherapy.

DUSP1(dual specificity phosphatase 1) is a member of the DUSP family. DUSP proteins are involved in the regulation of cellular plasticity cells and melanoma drug resistance and are potential targets for treatment of MAPKi-resistant melanoma (42). Also, DUSP1 is a critical negative regulator of the immune response and mediates the expression of inflammatory and anti-inflammatory factors (43). CXCL13(C-X-C motif chemokine ligand 13) is vital for the recruitment of B cells and T follicular helper cells into the tumor microenvironment. It is a potent chemoattractant cytokine that promotes the migration of cells expressing its cognate receptor, CXCR5. The CXCL13-CXCR5 axis has very important roles in immunotherapy for melanoma, especially in the combination of anti-PD1 therapy (44, 45). SLAMF7(signaling lymphocytic activation molecule family member 7) was reported to play an important role in modulating T cell function in the TME (46). As a receptor of Natural Killer (NK) cells, SLAMF7 tends to regulate NK cell cytotoxicity. This highlighted its value in SLAM-based targeted immunotherapies (47). EVI2B (ecotropic viral integration site 2B) involved in positive regulation of granulocyte differentiation. Recent studies have confirmed its role in immune infiltration among multiple myeloma (48), osteosarcoma (49). More importantly, EVI2B was confirmed to increase CD8+ T cells over regulatory T cells and its expression correlated with multiple immunomodulatory genes including IFN-γ signature genes in melanoma (50). These results further suggest that radiotherapy-related genes have the potential to improve the response of melanoma patients to immunotherapy.

We validated the expression of DUSP1, SLAMF7 and EVI2B in human melanoma cell UACC62 and UACC257 after 8 Gy gamma-ray. CXCL13 is a chemokine for B cells, primarily expressed in immune cells such as T follicular helper cells, dendritic cells and macrophages. Therefore, we did not check its expression in our human melanoma cell model.

However, our study has some limitations. Firstly, the sample size retrieved from the database was limited. Also, the findings lack *in vivo* experimental validation. In our future work, based on the four key genes identified in this study, we will comprehensively investigate the impact of radiotherapy on the immune microenvironment and immunotherapy response in melanoma patients by constructing *in vivo* models and collecting relevant clinical samples. Despite these shortcomings, our preliminary findings offer meaningful and constructive insights into the role of radiotherapy in melanoma immunotherapy.

## 5 Conclusion

In summary, our study underscores the importance of constructing accurate prognostic models for radiotherapy in melanoma to enhance disease management and patient outcomes. The interplay between immune infiltration and radiotherapy highlights the need for integrating immunological parameters into prognostic assessments. Furthermore, the identification of key genes and their associated signaling pathways offers promising avenues for future therapies. Lastly, the regulatory roles of radiation in melanoma present potential opportunities for novel prognostic and therapeutic strategies, especially in immunotherapy. Future research should focus on validating these findings in clinical settings and exploring their translational applications in melanoma treatment.

## Data Availability

The original contributions presented in the study are included in the article/**Supplementary Material**. Further inquiries can be directed to the corresponding author/s.

## Supplementary material

Supplementary 1 The identification value of four key genes for radiotherapy in melanoma patients was analyzed. AUC: Area under Curve.

Supplementary 2 Dot plot displaying marker genes of each cell type.

Supplementary 3 Details of RT patterns of patients in TCGA-SKCM database.
