# Supplementary figures and images for: Research on the influence of radiotherapy-related genes on immune infiltration, immunotherapy response and prognosis in melanoma based on multi-omics
Source: Front Immunol. 2024 Dec 2;15:1467098. doi: 10.3389/fimmu.2024.1467098 (PMC11647020; doi:10.3389/fimmu.2024.1467098)

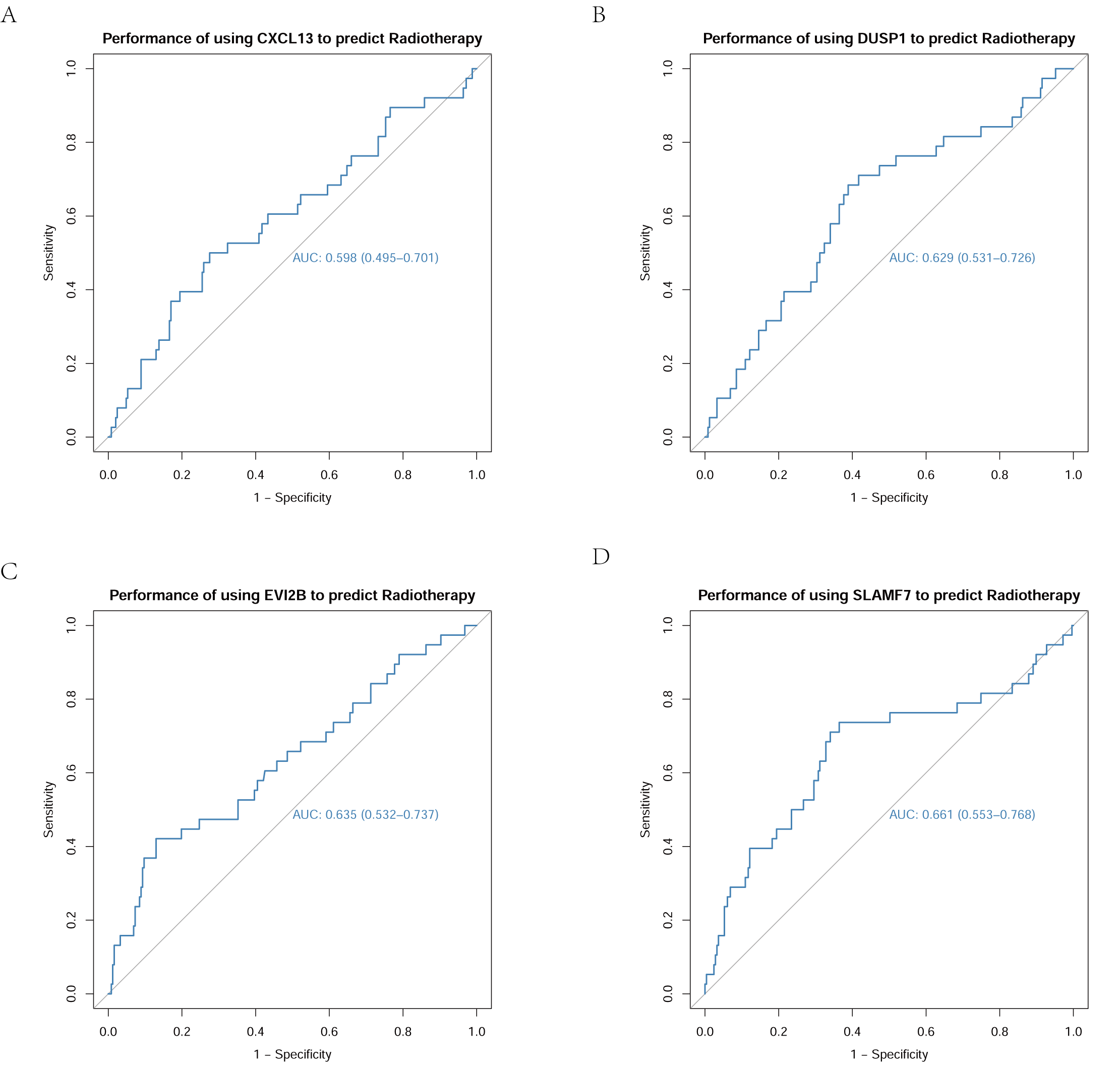

Supplement: Supplementary 1 — The identification value of four key genes for radiotherapy in melanoma patients was analyzed. AUC: Area under Curve. [file Image1.tif]

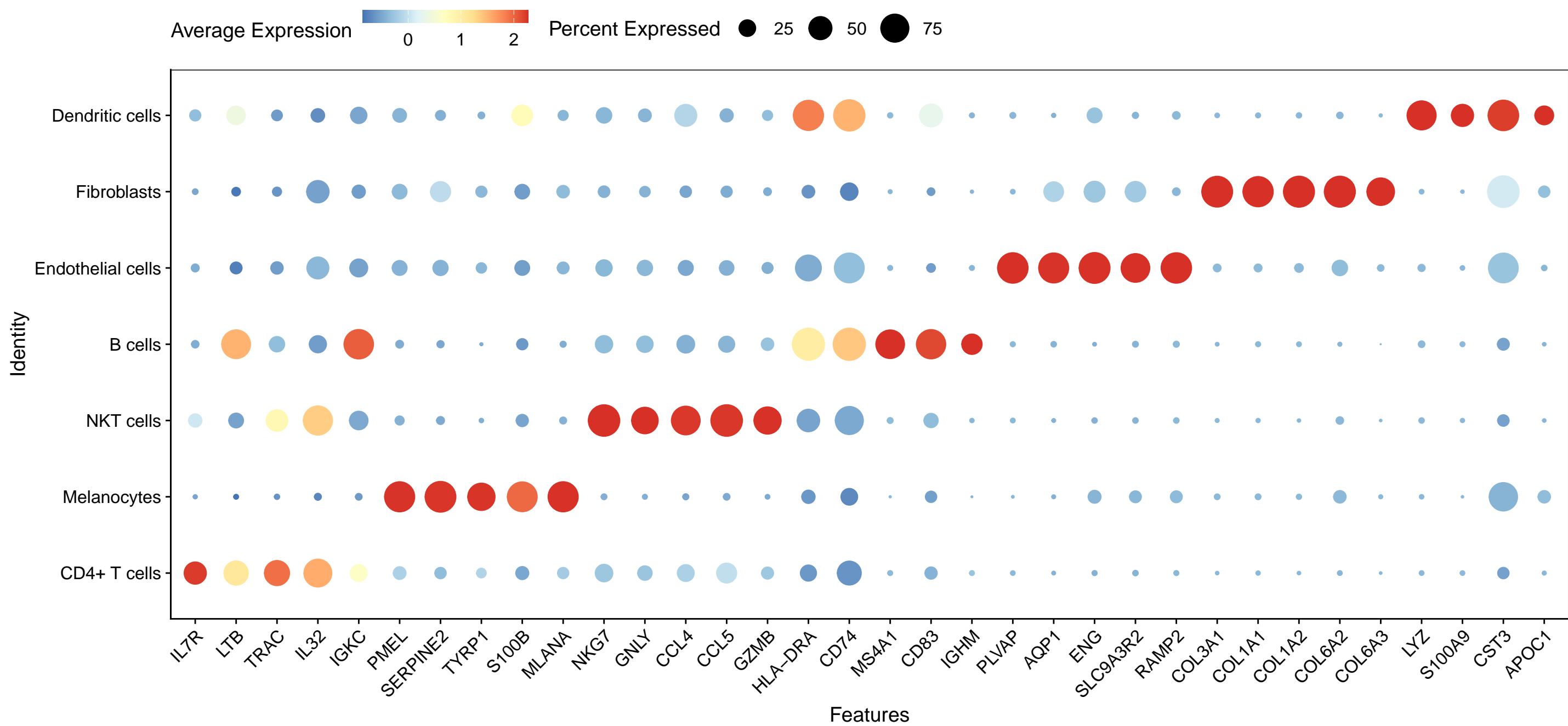

Supplement: Supplementary 2 — Dot plot displaying marker genes of each cell type. [file DataSheet1.pdf]
